# A randomised phase II trial of S-1 plus cisplatin versus vinorelbine plus cisplatin with concurrent thoracic radiotherapy for unresectable, locally advanced non-small cell lung cancer: WJOG5008L

**DOI:** 10.1038/s41416-018-0243-2

**Published:** 2018-09-12

**Authors:** Tomonari Sasaki, Takashi Seto, Takeharu Yamanaka, Naonobu Kunitake, Junichi Shimizu, Takeshi Kodaira, Makoto Nishio, Takuyo Kozuka, Toshiaki Takahashi, Hideyuki Harada, Naruo Yoshimura, Shinichi Tsutsumi, Hiromoto Kitajima, Masaaki Kataoka, Yukito Ichinose, Kazuhiko Nakagawa, Yasumasa Nishimura, Nobuyuki Yamamoto, Yoichi Nakanishi

**Affiliations:** 1 National Kyushu Cancer Center, 3-1-1 Notame, Minami-ku, Fukuoka, 811-1395 Japan; 2 Yokohama City University, 3-9 Fukuura, Kanazawa-ku, Yokohama, 236-0004 Japan; 3 Aichi Cancer Center Hospital, 1-1 Kanokoden, Chikusa-ku, Nagoya, 464-8681 Japan; 4 Cancer Institute Hospital of Japanese Foundation for Cancer Research, 3-8-31, Ariake, Koto-ku, Tokyo, 135-8550 Japan; 5 Shizuoka Cancer Center, 1007 Shimonagakubo, Nagaizumi-cho, Sunto-gun, Shizuoka, 411-8777 Japan; 6 Osaka City University Hospital, 1-5-7 Asahi-machi, Abeno-ku, Osaka, 545-8586 Japan; 7 Shikoku Cancer Center, 160 Kou, Minamiumemoto-machi, Matsuyama City, Ehime 791-0280 Japan; 8 Kindai University Hospital, 377-2 Ohnohigashi, Osaka-Sayama, 589-8511 Japan; 9 Kyushu University Hospital, 3-1-1 Maidashi, Higashi-ku, Fukuoka, 812-8582 Japan

**Keywords:** Non-small-cell lung cancer, Radiotherapy

## Abstract

**Background:**

Cisplatin-based chemoradiotherapy is the standard treatment for unresectable, locally advanced non-small-cell lung cancer (NSCLC). This trial evaluated two experimental regimens that combine chemotherapy with concurrent radiotherapy.

**Methods:**

Eligible patients with unresectable stage III NSCLC were randomised to either the SP arm (S-1 and cisplatin) or VP arm (vinorelbine and cisplatin), with early concurrent thoracic radiotherapy of 60 Gy, comprising 2 Gy per daily fraction. The primary endpoint was the overall survival rate at 2 years (2-year overall survival (OS)) (Study ID: UMIN000002420).

**Results:**

From September 2009 to September 2012, 112 patients were enroled. Of the 108 eligible patients, the 2-year OS was 75.6% (80% confidence interval (CI), 67–82%) in the SP arm and 68.5% (80% CI: 60–76%) in the VP arm. The hazard ratio (HR) for death between the two arms was 0.85 (0.48–1.49). The median progression-free survival was 14.8 months for the SP arm and 12.3 months for the VP arm with an HR of 0.92 (0.58–1.44). There were four treatment-related deaths in the SP arm and five in the VP arm.

**Conclusions:**

The null hypotheses for 2-year OS were rejected in both arms. The West Japan Oncology Group will employ the SP arm as the investigational arm in a future phase III study.

## Introduction

Non-small-cell lung cancer (NSCLC) is one of the leading causes of cancer death worldwide.^1^ The standard treatment for patients with unresectable, locally advanced NSCLC (LA-NSCLC) is chemotherapy with concurrent radiotherapy, although the optimal chemotherapeutic agents remain undefined. Recently, chemotherapeutic regimens have advanced from the second to third generation.^2,3^

Yamamoto et al.^3^ published the results of a phase III trial (WJTOG0105) that was conducted to compare third-generation chemotherapy (carboplatin with paclitaxel, or cisplatin with irinotecan) with second-generation chemotherapy (cisplatin plus mitomycin plus vindesine: MVP) both in conjunction with concurrent thoracic radiotherapy (TRT), and reported no significant difference in overall survival (OS). Segawa et al.^2^ also published the results of a phase III clinical trial that compared concurrent TRT with docetaxel plus cisplatin (DP) with concurrent TRT with MVP. There was a trend towards improved survival in the DP arm compared with the MVP arm (*p* = 0.059). However, these previous trials failed to show that third-generation regimens were significantly superior to second-generation regimens.

Alternatively, the West Japan Oncology Group (WJOG) has developed another chemotherapeutic regimen consisting of cisplatin and S-1 for patients with LA-NSCLC. S-1 (TS-1^®^; Taiho Pharmaceutical Co. Ltd., Tokyo, Japan) is a third-generation oral fluoropyrimidine anticancer agent that combines tegafur, gimeracil, and oteracil potassium in a molar ratio of 1.0:0.4:1.0.^4^ We conducted a single-arm phase II study, WJTOG3706, to evaluate the efficacy and safety of SP with concurrent TRT. The OS rate at 2 years (2-year OS) was 70% with a response rate of 84%. These are promising results and appear to be superior to other second-generation or third-generation regimens employed in previous clinical trials.^5^

At present, in conjunction with concurrent TRT, vinorelbine plus cisplatin (VP), or DP and carboplatin plus paclitaxel are third-generation regimens frequently used in patients with unresectable stage III LA-NSCLC. Radiotherapy was given concurrently during the second and third cycles of chemotherapy (four cycles in total) in overseas phase II studies, with reports that the median survival time ranged from 16 to 17 months.^6,7^ In Japan, Sekine et al.^8^ reported one-week cessation of ongoing radiotherapy and use of docetaxel as additional chemotherapy following concurrent radiotherapy using the VP regimen. However, no prospective studies have been conducted to evaluate concurrent radiotherapy and additional chemotherapy in a single VP regimen without suspending radiotherapy. Moreover, unlike SP regimens, no reproducible data are available regarding VP regimens. To select the optimal VP regimen in Japan, we therefore considered it necessary to give concurrent radiotherapy and additional chemotherapy in a single VP regimen on the basis of the regimen proposed by Sekine et al.,^8^ followed by an evaluation of this therapeutic approach.

As a result of the preceding study (WJTOG0105), carboplatin plus paclitaxel is now considered a standard chemoradiotherapy regimen for LA-NSCLC in Japan. However, cisplatin plus etoposide is standard chemoradiotherapy outside Japan, and a direct comparison with the cisplatin-based regimen is required. To evaluate which treatment regimen, SP or VP, would be best as an investigational arm in a future study, we decided to conduct a phase II randomised study and compare these regimens.

## Patients and methods

### Patient selection

Patients with histologically or cytologically proven NSCLC with unresectable, locally advanced disease were assessed for eligibility. The definition of locally advanced disease is described in detail in the protocol, although in brief it constitutes unresectable stage III disease. Eligible patients also needed to meet the following criteria: no prior history of chemotherapy or TRT or surgery; and patients who can be treated with radiotherapy according to the protocol. Other eligibility requirements included age of 20–74 years, Eastern Cooperative Oncology Group performance status of 0–1, and adequate organ function.

For staging, all patients underwent chest X-ray (CXR), computed tomography (CT) of the thorax and abdomen, and either brain CT or brain magnetic resonance imaging. A radioisotopic bone scan or positron emission tomography was also performed on all patients.

All patients provided written informed consent before enrolment in this study. The protocol was designed in accordance with the Declaration of Helsinki and ethical guidelines for clinical research and was approved by the institutional review boards at all participating institutions.

### Randomisation

Eligible patients were randomly assigned to the SP arm or the VP arm at the WJOG data centre. Randomisation was achieved by a minimisation method and stratified by disease stage (stage IIIA or IIIB), sex (male or female), histology (adenocarcinoma or non-adenocarcinoma) and institution. Patients and investigators were not masked to treatment.

### Procedures

Treatment comprised concurrent chemoradiotherapy and subsequent consolidation chemotherapy.

**Fig. 1:**
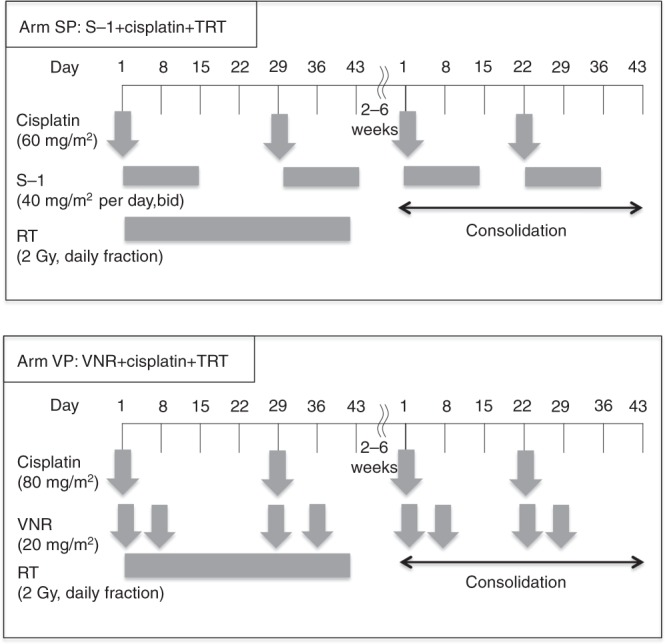
Treatment schedule. The treatment schedule of each arm. SP arm: S-1 plus cisplatin plus thoracic radiotherapy (TRT), VP arm: vinorelbine (VNR) plus cisplatin plus thoracic radiotherapy

In the SP arm, patients received oral S-1 (80 mg/m^2^) in two divided doses daily after meals on days 1–14 and cisplatin (60 mg/m^2^) as an intravenous (i.v.) infusion on day 1. The dose of S-1 was determined according to body surface area (BSA) as follows: BSA <1.25 m^2^, 80 mg per day; BSA 1.25 m^2^ to <1.50 m^2^, 100 mg per day; and BSA 1.5 m^2^ or higher, 120 mg per day. Combination chemotherapy with SP was repeated twice, with a 4-week interval, concurrently with TRT. In the VP arm, patients received vinorelbine 20 mg/m^2^ on days 1 and 8, and cisplatin 80 mg/m^2^ on day 1. Chemotherapy with VP was repeated every 4 weeks for two cycles, concurrently with TRT. Two to six weeks after the completion of the concurrent TRT, two further cycles of the same SP or VP regimen were administered every 3 weeks as consolidation chemotherapy (Fig. 1).

### Radiotherapy

All patients were treated with a linear accelerator photon beam of 6–10 MV from day 1. The primary tumour and involved nodal disease received 60 Gy in 2 Gy fractions over a period of 6 weeks. In this protocol, three-dimensional (D) treatment planning systems were acquired, and 40 Gy of prophylactic mediastinal irradiation was administered. The doses were calculated assuming tissue homogeneity with correction for lung tissue. The initial 40 Gy/20 fractions were delivered to clinical target volume 1 (CTV1), and the final 20 Gy/10 fractions were given to a reduced volume defined as clinical target volume 2 (CTV2). CTV1 included the primary tumour, ipsilateral hilum, and mediastinal nodal areas from the paratracheal (no. 2) to subcarinal lymph nodes (no. 7). For the primary tumours and involved lymph nodes with a short-axis diameter of 1 cm or larger, a margin of at least 0.5 cm was added. The contralateral hilum was not included in CTV1. The supraclavicular areas were not treated routinely, but were treated when the supraclavicular nodes were involved. CTV2 included only the primary tumour and the involved lymph nodes, with a margin of 0.5 to 1 cm. The spinal cord was excluded from the fields for CTV2 by appropriate methods, such as the oblique opposing method. An appropriate planning target volume margin and leaf margin were added for CTV1 and CTV2.

TRT was interrupted at the onset of grade 4 haematologic toxicity, grade 3 to 4 oesophagitis or dermatitis, pyrexia of 38 °C, or a decrease in the partial pressure of arterial oxygen of 10 Torr or more, compared with that measured before the initiation of TRT. If a rest period of more than 2 weeks was required, the patient was withdrawn from the study.

In this trial, radiotherapy data for all patients were submitted to the WJOG data centre and reviewed by the members of the radiotherapy committee (RC). The quality assurance (QA) assessment is described in the protocol. Individual cases were reviewed at a QA review meeting, which was held after each cohort of 15–20 patients had been registered. All data items including pretreatment images (contrast-enhanced chest CT, ^18^F-fluorodeoxyglucose positron emission tomography/CT) and radiotherapy data (beam data, target volume, dose distribution, dose–volume histogram) submitted to the data centre were reviewed and evaluated in the meeting.

### Evaluation of efficacy and toxicity

All eligible patients who received any treatment at all were considered assessable for response and toxicity. CXR, complete blood counts and blood chemistry tests were repeated once a week during the treatment period. Thoracic CT was performed once a month during the treatment period. After the conclusion of treatment, thoracic CT was performed every 3 months and other imaging examinations were performed when recurrence was suspected. Responses ≥were evaluated according to Response Evaluation Criteria in Solid Tumour, version 1.0. During evaluation of the response, extramural review was conducted. Adverse events were evaluated according to the Common Terminology Criteria for Adverse Events (v3.0). OS was defined as the time from registration until death from any cause. Progression-free survival (PFS) was defined as the time between enrolment and disease progression, death or last known follow-up.

### Statistical analysis

The full analysis set included all patients who received the study treatment at least once, were observed for survival, and did not violate the eligibility criteria. The safety analysis set was defined as all patients who received the study treatment at least once. The primary endpoint of this trial was comparison of the 2-year OS rate between the SP arm and VP arm at 2 years (2-year OS rate). This trial was designed to test the null hypothesis that the true 2-year OS rate is less than or equal to a threshold of 50% versus the alternative hypothesis that the true 2-year OS rate is ≥65%. With this design, the one-sided *α* is 0.10. The 80% two-sided confidence interval (CI) for 2-year OS rate was used to test the null hypothesis. The CI was calculated using Greenwood’s formula. The patient assignment period was 2 years, and the follow-up period was 2 years. In view of the possibility of variance inflation owing to censoring, the sample size was set at 110. Baseline characteristics were compared among the treatment groups using the Kruskal–Wallis test for continuous variables and Fisher’s exact test for discrete variables. Rates of specific toxicities and treatment delivery were compared between the groups using Fisher’s exact test. Survival curves were estimated by the Kaplan–Meier method. The hazard ratios (HRs) were estimated using the Cox proportional hazards method. All statistical analyses were performed using the SAS version 9.1.3 software. We also evaluated OS, PFS, treatment completion rate, and safety as secondary endpoints.

## Results

Four treatment-related deaths (TRDs) were reported in the period between December 2009 and February 2011, and these occurred up to the assignment of the seventy-third patient. Therefore, we suspended registration and checked the details of all randomised patients to assess the safety of treatment regimens. The WJOG Data and Safety Monitoring Committee advised consultation with the WJOG RC about radiotherapy compliance in all patients. The WJOG RC collected each patient’s irradiation planning data and did not find an association between poor protocol compliance and TRD. However, it was found that patients who died of radiation pneumonitis had interstitial changes on pretreatment chest CT but not on CXR. Consequently, in August 2011 we decided to continue this trial following the recommendations of the WJOG DSMC, in which the exclusion criteria were updated to exclude patients who had interstitial pneumonia, pulmonary fibrosis or severe emphysema on chest CT images, or who had obstructive pneumonia, active infection (e.g. those who had a fever of at least 38 °C and who used non-steroidal anti-inflammatory drugs to treat fever), and other serious complications (such as gastrointestinal haemorrhage and cardiac disease).

### Patient characteristics

Between September 2009 and September 2012, a total of 112 patients were registered for the study, and 56 patients were allocated to each arm. Of the total, four patients did not receive the protocol treatment because they became ineligible based on the protocol criteria after registration (two patients) or refused to participate (two patients) (Fig. S1).

The safety and antitumour effects of the treatments were eventually assessed on the basis of the data obtained from 108 enroled patients. There were no statistically significant differences between the arms in terms of patient characteristics (Table 1).

**Table 1:** Baseline demographic and patients characteristics

	SP (*n*=54)	VP (*n*=54)	*P* value
Gender, *n* (%)			1
Male	42 (77.8)	43 (79.6)	
Female	12 (22.2)	11 (20.4)	
Age, median (range)	60 (39–73)	62 (37–74)	0.073
Stage, *n*(%)			1
IIIA	22 (40.7)	22 (40.7)	
IIIB	32 (59.3)	32 (59.3)	
Primary site			0.478
Right upper lobe	28 (51.9)	21 (38.9)	
Right middle lobe	0 (0.0)	2 (3.7)	
Right lower lobe	2 (3.7)	3 (5.6)	
Left upper lobe	18 (33.3)	23 (42.6)	
Left lower lobe	3 (5.6)	4 (7.4)	
Others	3 (5.6)	1 (1.9)	
Histology, *n* (%)			0.92 (adeno versus non-adeno)
Adenocarcinoma	30 (55.6)	30 (55.6)	
Squamous cell carcinoma	17 (31.5)	17 (31.5)	
Adenosquamous cell carcinoma	1 (1.9)	0 (0.0)	
Large-cell carcinoma	2 (3.7)	1 (1.9)	
Others	4 (7.4)	6 (11.1)	
Smoking status, *n* (%)			1
Never	6 (11.1)	7 (13.0)	
Ever	48 (88.9)	47 (87.0)	
PS, *n* (%)			0.694
0	34 (63.0)	31 (57.4)	
1	20 (37.0)	23 (42.6)	
Complication, *n* (%)			0.335
Absent	29 (53.7)	23 (42.6)	
Present	25 (46.3)	31 (57.4)	
Lung V20 (%), median (range)	23.5 (13.1–34.7)	25.2 (11.4–34.7)	0.306

*SP* cisplatin plus S-1, *VP* cisplatin plus vinorelbine, *PS* performance status

### Treatment administered

During the concurrent phase, 70.4% of patients in the SP arm and 48.1% in the VP arm received full cycles of radiotherapy and chemotherapy without any dose reduction within the phase (*p* = 0.031). Radiotherapy of 60 Gy was completed in 102 patients. The main reasons for radiotherapy interruption in all treated patients were fever (9.3%) in the SP arm and fever (43.4%), neutropenia (32.1%), and leukopenia (24.5%) in the VP arm. In the consolidation phase, 59.3% and 44.4% in the SP arm and the VP arm, respectively, received the two scheduled courses of therapy. Interruption of chemotherapy was more common in the VP arm than in the SP arm during both the concurrent and consolidation phases. Of all treated patients, 7.4% of patients in the SP arm had a chemotherapy dose reduction, predominantly because of neutropenia (3.7%), thrombocytopenia (3.7%), and non-haematologic toxicities (3.7%) and 37.0% of patients in the VP arm had a chemotherapy dose reduction, predominantly because of neutropenia (27.7%), leukopenia (16.6%), and non-haematologic toxicities (14.8%). Overall, the treatment completion rates were 51.9% and 29.6% in the SP and VP arms, respectively (*p* = 0.031) (Table S1).

### Toxicity

Table 2 shows the all-grade and severe toxicities (grades 3–5) including nine patients with TRDs. With regard to grade 3–5 toxicities, leukopenia, neutropenia, and febrile neutropenia were more common in the VP arm than in the SP arm. Alternatively, grade 3–4 thrombocytopenia, oesophagitis, and diarrhoea tended to be more common in the SP arm than in the VP arm.

**Table 2:** Treatment-related toxicities

Adverse events, *n* (%)	SP (*n*=54)		VP (*n*=54)	
	All grades	Grade 3–5	All grades	Grade 3–5
Haematologic toxicities				
Leukopenia	52 (96.3)	22 (40.7)	54 (100.0)	43 (79.6)
Neutropenia	48 (88.9)	18 (33.3)	51 (94.4)	41 (75.9)
Thrombocytopenia	23 (42.6)	5 (9.3)	12 (22.2)	2 (3.7)
Anaemia	43 (79.6)	14 (25.9)	48 (88.9)	15 (27.8)
Febrile neutropenia	5 (9.3)	5 (9.3)	9 (16.7)	9 (16.7)
Non-haematologic toxicities				
AST increase	15 (27.8)	0 (0.0)	15 (27.8)	2 (3.7)
ALT increase	23 (42.6)	0 (0.0)	25 (46.3)	4 (7.4)
Total bilirubin increase	14 (25.9)	0 (0.0)	8 (14.8)	0 (0.0)
Nausea	42 (77.8)	2 (3.7)	41 (75.9)	2 (3.7)
Vomiting	10 (18.5)	1 (1.9)	13 (24.1)	0 (0.0)
Anorexia	46 (85.2)	7 (13.0)	48 (88.9)	8 (14.8)
Fatigue	37 (68.5)	2 (3.7)	39 (72.2)	2 (3.7)
Oesophagitis	36 (66.7)	2 (3.7)	40 (74.1)	0 (0.0)
Mucositis	10 (18.5)	0 (0.0)	9 (16.7)	0 (0.0)
Diarrhoea	19 (35.2)	3 (5.6)	9 (16.7)	0 (0.0)
Creatinine increase	15 (27.8)	0 (0.0)	28 (51.9)	0 (0.0)
Hyponatremia	39 (72.2)	10 (18.5)	35 (64.8)	4 (7.4)
Pneumonitis	13 (24.1)	5 (9.3)	11 (20.4)	4 (7.4)
Bleeding	1 (1.9)	1 (1.9)	2 (3.7)	2 (3.7)
Injection site reaction	1 (1.9)	0 (0.0)	13 (24.1)	0 (0.0)

*AST* aspartate aminotransferase, *ALT* alanine aminotransferase, *SP* cisplatin plus S-1, *VP* cisplatin plus vinorelbine

The cause of TRD was radiation pneumonitis in three patients and bleeding in one of the four patients in the SP arm: radiation pneumonitis in one patient, pneumonia in two patients, and bleeding in two of the five patients in the VP arm. The clinical course of the patients who died of pneumonitis and bleeding are presented in detail in the Supplementary results. QA results from radiotherapy of all patients showed neither improper protocol implementation nor deviation was directly associated with TRDs.

**Fig. 2:**
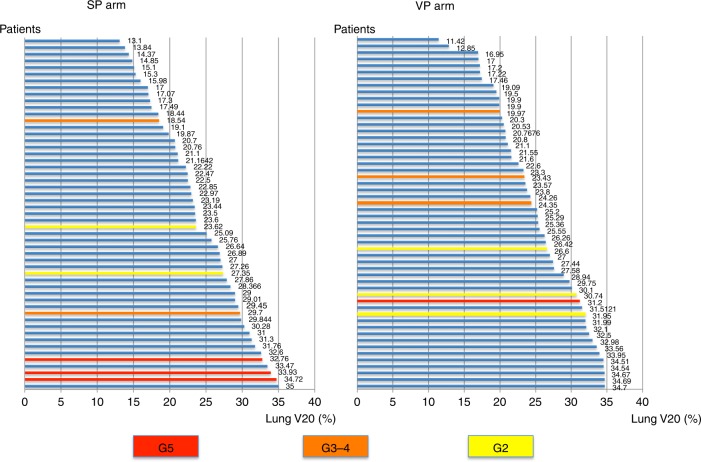
Relationship between lung V20 and radiation pneumonitis. The relationship between lung V20 and pneumonitis grading in the SP arm (S-1 plus cisplatin plus thoracic radiotherapy) and the VP arm (vinorelbine plus cisplatin plus thoracic radiotherapy). The patients are arranged in ascending order of V20 on the vertical axis and lung V20 is plotted on the horizontal axis; the grade of pneumonitis is colour coded

The relationship between the lung V20 measurement and the grade of radiation pneumonitis is shown in Fig. 2. The risk of radiation pneumonitis grades 2–5 was related to a V20 of 30% or more (*p* = 0.048).

### Efficacy

**Fig. 3:**
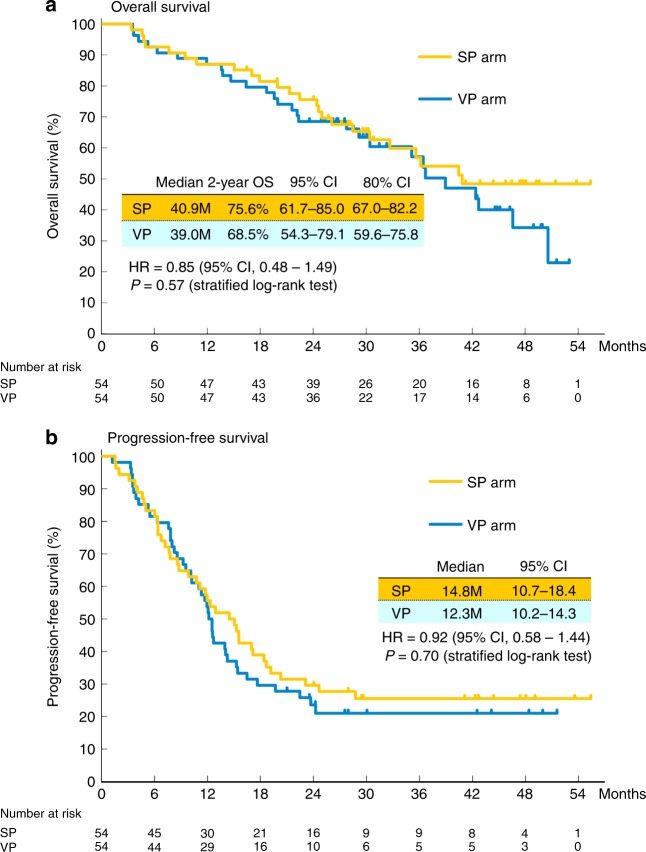
Survival curves. Kaplan–Meier estimates of **a** overall survival (OS; primary endpoint) in the full analysis set (FAS) and **b** progression-free survival (PFS) in the FAS. OS was measured from the date of random assignment to the date of death as a result of any cause. At the cutoff date for data inclusion in the analysis, if a patient had not died, OS was censored at the last date they were known to still be alive. PFS was measured from the date of random assignment to the first date of documented objective progression of disease, or of death as a result of any cause. PFS was censored at the date of the last objective progression-free disease assessment before the date of any subsequent systemic anticancer therapy or death, whichever applied first. CI confidence interval, SP arm S-1 plus cisplatin plus thoracic radiotherapy, VP arm vinorelbine plus cisplatin plus thoracic radiotherapy

**Fig. 4:**
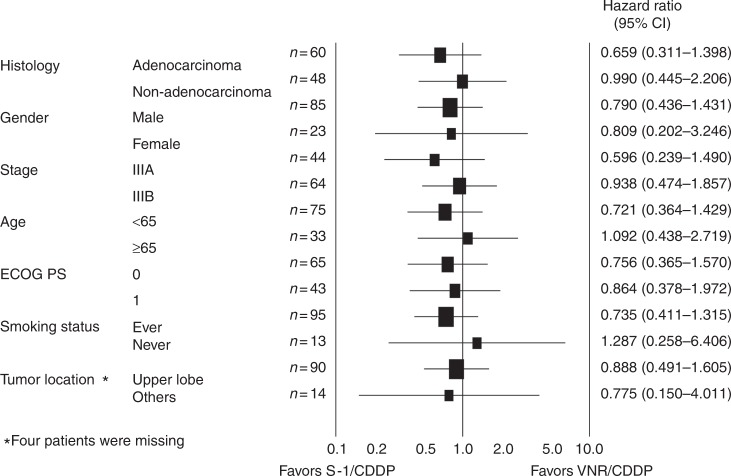
Subset analysis. Overall survival hazard ratio in subgroups according to baseline characteristics. CI confidence interval, ECOG PS Eastern Cooperative Oncology Group performance status, CDDP cisplatin, VNR vinorelbine

The objective response rates were 76.9% (95% confidence interval (CI): 63.2–87.5) and 80.8% (95% CI: 67.5–90.4) in the SP and VP arms, respectively (Table S2). No efficacy data were obtained from two patients in both arms and all of these results are based on the analysis of 52 patients. There was no statistical difference between these arms. The OS and PFS curves are shown in Fig. 3. Most of the patients were observed for more than 2 years, and 52 patients died. The median follow-up time for the censored patients was 31.9 months (interquartile range: 27.7–47.7 months), and the 2-year PFS rate for all treated patients was 26.7% (95% CI: 18–35%). The median survival time, the median PFS time, and 2-year OS in the SP arm were 40.9 months, 14.8 months, and 75.6% (80% CI: 67–82%), respectively. The corresponding values in the VP arm were 39.0 months, 12.3 months, and 68.5% (80% CI: 60–76%). The lower limit of the CI for 2-year OS in both arms exceeded the threshold of 50%. However, there was no statistically significant difference in terms of OS between the two arms (HR = 0.85; 95% CI: 0.48–1.49; *p* = 0.57). Subset analyses show that the OS was not significantly different when various factors are taken into consideration (Fig. 4). There was also no statistically significant difference in PFS between the arms (HR = 0.92; 95% CI: 0.58–1.44; *p* = 0.70).

### Pattern of recurrence

In this trial, disease recurred in 35 patients in the SP arm and 38 patients in the VP arm. Sites of initial recurrence are shown in Table S3 stratified by the arms. Among the 35 patients with recurrence in the SP arm, in-field relapse was observed in 17 patients (49%, 12 without and 5 with relapse outside of radiation fields). Distant metastases were the first site of failure in 23 patients. Meanwhile, among the 38 patients with recurrence in the VP arm, in-field relapse was observed in 28 patients (74%, 19 without and 9 with relapse outside of radiation fields). Distant metastases were the first site of the failure in 19 patients. After the first relapse, 25 and 30 patients in the SP arm and VP arm, respectively, received second-line or further chemotherapy.

## Discussion

This randomised phase II trial was undertaken to evaluate two chemotherapy regimens (SP versus VP) concurrently performed with TRT in patients with unresectable LA-NSCLC. No significant difference between the arms was found. The levels of efficacy and toxicity in each arm were comparable to those seen previous phase II trials. The null hypothesis was rejected in both arms, suggesting that the findings of the present study may be useful in conducting a WJOG-sponsored phase III study, with carboplatin plus paclitaxel as the standard of care. However, in the present study, the frequency of TRDs due to radiotherapy was slightly higher than expected, while the QA results from radiotherapy did not indicate that deviations were directly associated with TRDs. Thus, we need to review exclusion criteria for radiotherapy to provide safer treatment for patients with LA-NSCLC in the future trial.

The present study also adopted the regimen used in WJTOG3706 for the SP arm. Ohyanagi et al.^9^ and Kaira et al.^10^ have also conducted phase II studies using SP regimens and the efficacy of the SP regimen used in the present study is similar to that reported in their studies; the toxicity is considered acceptable. Various VP regimens have been examined (i.e. no radiation in the first cycle and concurrent radiotherapy in the second and third cycles with additional chemotherapy in the fourth cycle, or oral administration of vinorelbine) and no treatment strategy has been established for this regimen.^6,7,11,12^ Induction chemotherapy is no longer considered to be the standard of care for lung cancer. We thus adopted the regimen proposed by Sekine et al.,^15^ which is the most common chemoradiotherapeutic regimen used in Japan. Sekine et al.^8,15^ used 1-week cessation of radiation during treatment. However, recent radiotherapy is, in principle, given without cessation and additional adjuvant chemotherapy generally uses the same regimen as that used in concurrent radiotherapy. Therefore, radiotherapy was continued without cessation in this study and the regimen prescribed for concurrent radiotherapy was used instead of docetaxel during the consolidation phase. Although the efficacy of the VP regimen studied was similar to those reported in previous studies, toxicity was higher than that expected and higher than that observed with other regimens using vinorelbine. However, we consider the toxicity of our VP regimen to be acceptable, suggesting that the regimen used in the present study could become a standard VP regimen.

When these results are compared with those from previous phase III studies, 2-year PFS is equal to that reported in PROCLAIM, RTOG0617, and WJTOG0105 and toxicity is acceptable, except for a slightly higher incidence of TRDs;^3,13,14^ both the SP and VP regimens should be fully evaluated as potential treatments in future studies.

There was no difference in the treatment completion rate between the VP arm and the SP arm during the consolidation phase, although the VP arm had a lower treatment completion rate than the SP arm during the concurrent phase for several reasons including delayed treatment and discontinuation due to haematological toxicity. The treatment completion rate in the SP arm was similar to that reported in WTOG3706 and other phase II studies, and the obtained results may be reproducible. Because there were variations in previous VP regimens, this made direct comparison somewhat difficult.^6–8,11^ However, haematological toxicity tended to be higher in the VP arm than in the SP arm and treatment suspension and dose reduction were required. Although efficacy and toxicity appeared to be similar in the two arms, the SP arm tended to have slightly more favourable OS results in every subgroup, and PFS in the SP arm also appeared to be superior to that in the VP arm for a long-term course of treatment (Fig. 3a). This may be due to the fact that the SP arm has a slightly better outcome in terms of local control and treatment compliance.

The patterns of first failure by site were different between the arms. A preceding phase II study (WJTOG3706) also showed that distant failure was more frequent than local failure (68% versus 32%).^5^ Other previous studies have also reported that the incidence of recurrence within the radiation field tended to be low in the SP regimen and no specific tendency was observed for the VP regimen.^9,10,15,16^ The risk of local recurrence may be reduced by continuous administration of S-1 acting as a radiosensitizer in the SP regimen. In contrast, the frequency of distant recurrence is high, and it may be necessary to evaluate additional regimens which are more effective against distant metastases.

More TRDs occurred in this trial than were expected, although all of the adverse events reported in this series can occur with TRT for lung cancer. A previous phase I/II trial for patients with lung cancer treated with combination TRT and SP regimens or VP regimens showed a TRD rate of <5%.^5,9–12,16–21^

Three patients in the SP arm and one patient in the VP arm died of radiation pneumonitis. Palma et al.^22^ reported that fatal pneumonitis, although uncommon, is related to dosimetric factors such as V20 and mean lung dose. In the present study, the frequency of radiation pneumonitis (grade 2 or greater) was significantly increased in patients with a V20 ≥30%, which is also consistent with the report. Although previous studies (PROCLAIM, RTOG0617, KCSG-LU05-04) have reported that the risk of TRDs, including fatal radiation pneumonitis and pulmonary haemorrhage, is 3% to 5%, the rate was relatively high in the present study at 8%.^13,14,23^ QA results from radiotherapy in all patients indicated that there were no TRDs caused by deviations from the protocol. There was no significant relationship between V20 >30% and radiation pneumonitis grade 5, although the lung V20 exceeded 30% in patients in the SP arm who died of radiation pneumonitis. Regarding pulmonary doses (V20), regulatory controls should be stricter in future clinical trials

The present study has the following limitations: (1) as with previous studies, the significance of consolidation is not clear; (2) the VP regimen adopted may not have been optimal; and (3) the study population was exclusively Japanese and the obtained data cannot be regarded as globally applicable.

Although the present study found no significant difference between the SP arm and the VP arm, the PFS curve showed more favourable results for the SP arm over the long term, and treatment compliance was better in the SP arm than in the VP arm. After a comprehensive consideration of these results, WJOG therefore intends to study the SP arm in the next phase. However, results for adjuvant treatment with durvalumab after definitive chemoradiotherapy for patients with LA-NSCLC have been reported recently.^24^ Consolidation chemotherapy using an immune checkpoint inhibitor may become standard treatment in the near future. Therefore, we have to evaluate the feasibility of consolidation chemotherapy using durvalumab after TRT with concurrent S-1 plus cisplatin while carefully considering the risk of pulmonary toxicity caused by radiotherapy before initiation of the phase III trial.

## Electronic supplementary material

Supplementary data summary

Supplementary results

Figure S1

Table S1

Table S2

Table S3

## Availability of data and material

All data are available via the corresponding author and the WJOG.
