# Supplementary material for: A randomised phase II trial of S-1 plus cisplatin versus vinorelbine plus cisplatin with concurrent thoracic radiotherapy for unresectable, locally advanced non-small cell lung cancer: WJOG5008L
Source: Br J Cancer. 2018 Sep 12;119(6):675–82. doi: 10.1038/s41416-018-0243-2 (PMC6173687; doi:10.1038/s41416-018-0243-2)
Supplement: Supplementary file 1 — Supplementary data summary [file 41416_2018_243_MOESM1_ESM.docx]

Summary of supplementary files

1) BJC_SupplementaryTable S1.xlsx

Radiotherapy and chemotherapy administered

2) BJC_SupplementaryTable S2.xlsx

Objective response rate of each regimen

3) BJC_SupplementaryTable S3.xlsx

First recurrent site of each regimen

4) SupplemenataryFigureS1.tif

Consort diagram：SP represents concurrent S-1 plus cisplatin and thoracic radiotherapy (TRT) followed by consolidation with S-1 plus cisplatin. VP represents concurrent vinorelbine plus cisplatin and TRT followed by consolidation with vinorelbine plus cisplatin. The first patient was randomly assigned on November 5, 2009.

5) Supplementary results.docx

Supplementary results of Treatment-related deaths, in detail
