# Supplementary material for: A randomised phase II trial of S-1 plus cisplatin versus vinorelbine plus cisplatin with concurrent thoracic radiotherapy for unresectable, locally advanced non-small cell lung cancer: WJOG5008L
Source: Br J Cancer. 2018 Sep 12;119(6):675–82. doi: 10.1038/s41416-018-0243-2 (PMC6173687; doi:10.1038/s41416-018-0243-2)
Supplement: Supplementary file 2 — Supplementary results [file 41416_2018_243_MOESM2_ESM.docx]

# Supplementary results of Treatment-related deaths, in detail

One patient (#48) in the VP arm had a large tumor in the right upper lobe, with a cavitary lesion with an air-fluid level on CT performed before treatment. He died of bleeding from the cavity two months after RT. This patient had chronic infection in the cavity before registration. We revised the protocol, so that in future a patient with repeated infection of the tumor could not be enrolled. One patient (#46) in the VP arm with necrosis in metastatic mediastinal lymph nodes after chemoradiothrapy had a fistula between the esophagus and mediastinum, and had mediastinitis. He died suddenly due to bleeding from an esophageal fistula two months after RT. One patient (#67) in the SP arm had G2 radiation pneumonitis which was controlled. He had a fistula between his right main bronchus and a necrotic right hilar lymph node, and died suddenly due to bleeding from the fistula.

In addition, four patients died of radiation pneumonitis. One patient (#35) in the VP arm developed Grade 2 radiation pneumonitis during consolidation chemotherapy. Treatment was ceased and the patient received pulsed steroid therapy, resulting in temporary improvement. He however ultimately died from an acute exacerbation of radiation pneumonitis. Another patient (#59) had radiation pneumonitis (G1) after consolidation chemotherapy. The pneumonitis temporarily improved, but he suddenly deteriorated and died. A further patient (#33) in the SP arm had G1 radiation pneumonitis during consolidation chemotherapy. Six months after the end of consolidation chemotherapy, he developed acute respiratory distress syndrome due to radiation pneumonitis, and died 14 months after the end of his final chemotherapy. One patient (#108) in the SP arm had radiation pneumonitis grade 1, one month after the final course of consolidation chemotherapy. Two month later, he suffered acute worsening of the pneumonitis. He then died of radiation pneumonitis despite methylprednisolone pulse therapy.

The other two patients died of pulmonary infection. One patient (#31) with adenocarcinoma developed a pulmonary infection after consolidation chemotherapy, and died 161 days after registration. The final patient (#83), in the VP arm, acquired pulmonary infection during concurrent chemoradiotherapy. He had repeated pulmonary infection culminating in a pulmonary abscess and death 111 days after registration.
