# Supplementary figures and images for: A randomised phase II trial of S-1 plus cisplatin versus vinorelbine plus cisplatin with concurrent thoracic radiotherapy for unresectable, locally advanced non-small cell lung cancer: WJOG5008L
Source: Br J Cancer. 2018 Sep 12;119(6):675–82. doi: 10.1038/s41416-018-0243-2 (PMC6173687; doi:10.1038/s41416-018-0243-2)

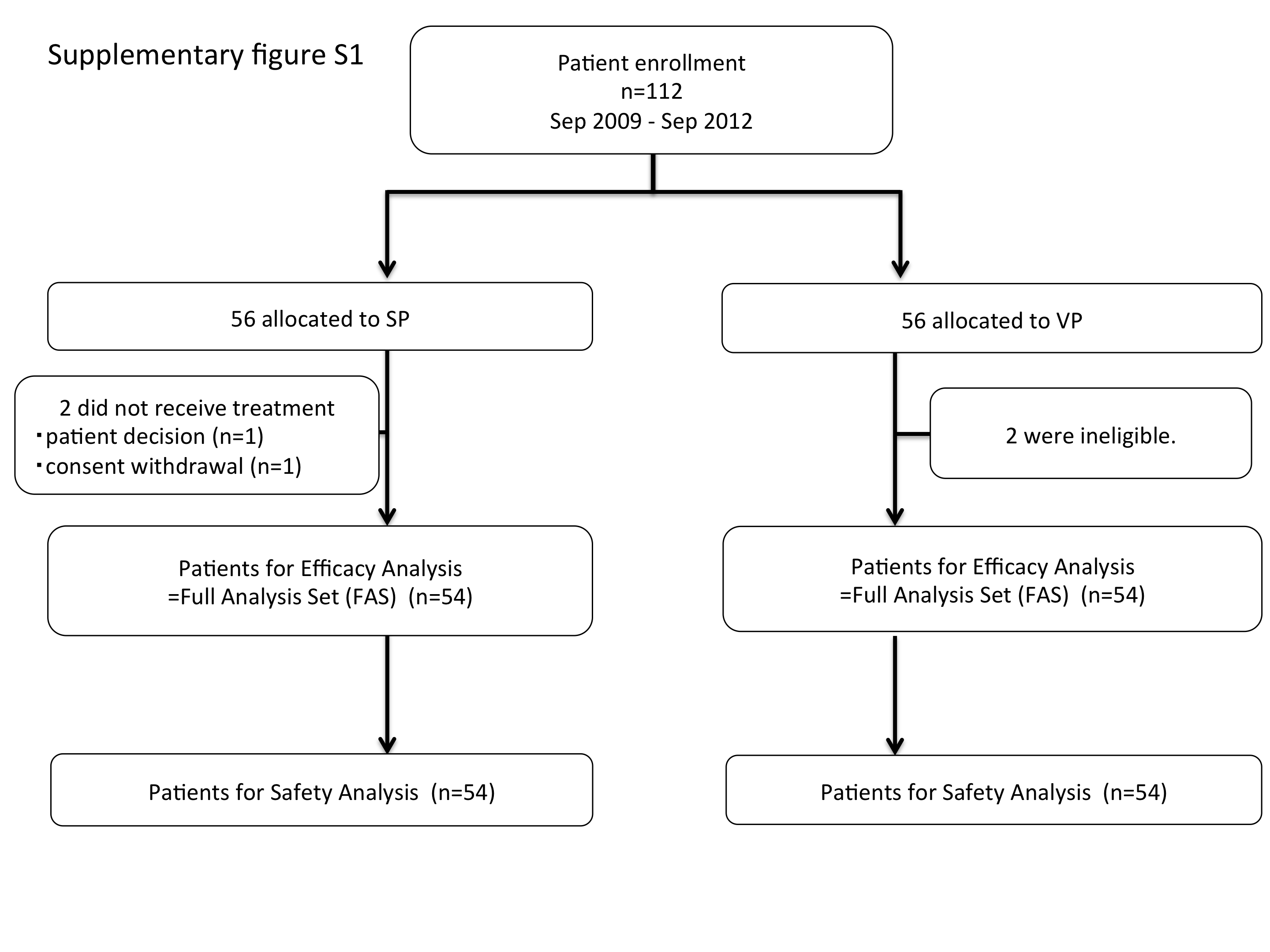

Supplement: Supplementary file 3 — Figure S1 [file 41416_2018_243_MOESM3_ESM.tif]
